# Meningococcal Group W Disease in Infants and Potential Prevention by Vaccination

**DOI:** 10.3201/eid2208.160128

**Published:** 2016-08

**Authors:** Sydel R. Parikh, Helen Campbell, Kazim Beebeejaun, Sonia Ribeiro, Steve J. Gray, Ray Borrow, Mary E. Ramsay, Shamez N. Ladhani

**Affiliations:** Public Health England, London, UK (S.R. Parikh, H. Campbell, K. Beebeejaun, S. Ribeiro, S.J. Gray, R. Borrow, M.E. Ramsay, S.N. Ladhani);; St. George’s University of London, London (S.N. Ladhani)

**Keywords:** meningococcal disease, MenW disease, vaccination, Bexsero, prevention, infants, United Kingdom, bacteria, vaccines, meningococci, group W

**To the Editor:** We recently reported that postvaccination serum samples from infants immunized with a novel, protein-based multicomponent meningococcal serogroup B (MenB) vaccine (Bexsero; GlaxoSmithKline Vaccines, Verona, Italy) have bactericidal activity against the hypervirulent meningococcal group W (MenW) strain belonging to the sequence type (ST) 11 clonal complex (*1*). Historically, MenW has been a rare cause of invasive meningococcal disease (IMD), accounting for <5% of confirmed cases in England and Wales (*2*). Since 2009, MenW cases caused by this hypervirulent strain have rapidly increased in England (*2*). During the 2014–15 epidemiologic year (July 1–June 30), this capsular group accounted for 176 (24%) of 724 IMD cases in England (*3*). In response to this outbreak, in August 2015, the United Kingdom introduced an emergency adolescent conjugate vaccination program against meningococcal capsular groups ACW and Y. Over 2 years, the program aims to provide vaccine to all youth 13–18 years of age and to new university entrants <25 years of age. This program is expected to protect adolescents (25 of 176 [14%] MenW cases during 2014–15 were in those 15–19 years of age), and, by targeting youth with the highest carriage rates, to protect others through indirect (herd) protection, which has been consistently observed in vaccine programs, including that for meningococcal group C (*4**,**5*). Indirect protection associated with the adolescent immunization program will likely take several years to manifest (*6*).

Infants <1 year of age have the highest incidence of IMD and the highest number of IMD cases and deaths (*5*). During the 2014–15, 127 (18%) of the 724 IMD cases in England occurred in this group: 101 (80%) meningococcal group B (MenB) cases, 21 (16%) MenW cases, 1 (1%) group C case, and 4 (3%) group Y cases (*7*). In September 2015, MenB vaccine was introduced into the UK infant immunization program under a 2-, 4-, and 12-month schedule. We analyzed the epidemiology and long-term trends for MenW disease in infants in England to assess the potential effects of the infant MenB immunization program for preventing MenW cases in this highly vulnerable age group.

During the epidemiologic period 1998–99 through 2014–15, a total of 176 MenW cases were confirmed in infants. The number of cases peaked during 2000–01 (n = 28) because of a national outbreak associated with the Hajj pilgrimage and then declined rapidly after mandatory vaccination for pilgrims was instigated (Figure, panel A). During that outbreak, most infants acquired the infection indirectly from family members who traveled to the Hajj, highlighting this group’s susceptibility to IMD. The number of MenW cases in infants began increasing again from 4 cases in 2012–13 to 12 in 2013–14 and 21 in 2014–15. During 2014–15, these 21 MenW cases represented 16.5% of 127 total IMD cases among infants, 12% of 176 total MenW cases, and 3% of 724 total IMD cases in England. All infants with MenW IMD resided in England and had not traveled abroad. The number of MenW cases increased from birth among infants, peaking at 4 months of age and remaining high until the first birthday. Most (123 [70%]) of the 176 MenW cases confirmed during (44/66 [67%]) and after (79/110 [72%]) the Hajj outbreak were in persons >5 months of age and were potentially preventable by MenB vaccine vaccination.

**Figure:**
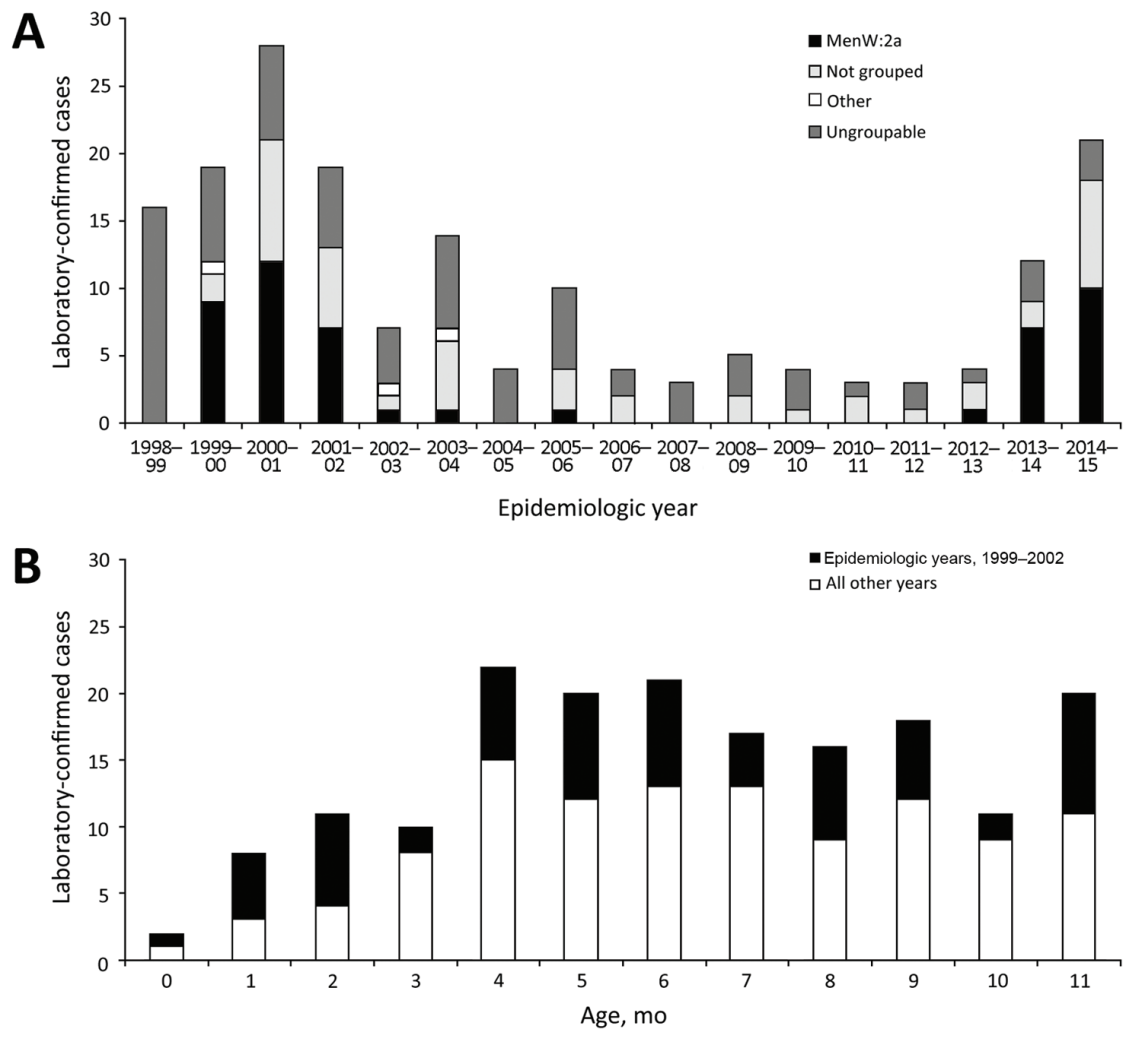
Incidence of invasive meningococcal disease (IMD) in infants <1 year of age in England during the epidemiologic years 1998–99 through 2014–15. A) Incidence of IMD and phenotypic characterization of laboratory-confirmed meningococcal group W strains in infants <1 year of age. B) Total laboratory-confirmed meningococcal group W cases in infants <1 year of age by month of age. Cases related to the Hajj outbreak occurred during 1999–00 through 2001–02.

During 2012–13 through 2014–15, a total of 25 (67.5%) of 37 MenW cases in infants were confirmed by culture; 18 (49%) of these cases were phenotypically characterized as MenW:2a, a surrogate phenotypic marker for the hypervirulent ST11 MenW strain. Ten (48%) of the 21 isolates from infants during 2014–15 were MenW:2a, compared with 1 (25%) of 4 during 2012–13 (Figure, panel B). Final diagnoses reported for 20 infants included meningitis (n = 10 [50%]), septicemia (n = 3 [15%]), both meningitis and septicemia (n = 5 [10%]), and septic arthritis (n = 1 [2%]). From 1998–99 through 2014–15, six infants died of MenW IMD (case-fatality rate 3.4%). Four of those deaths occurred during the Hajj outbreak; only 1 death attributed to MenW occurred during the 3 most recent epidemiologic years.

The rapid increase in MenW cases among infants, particularly most recently (2014–15), is cause for concern, and the contemporaneous introduction of MenB vaccine into the national immunization schedule is timely. Although this vaccine is licensed for prevention of MenB disease, the antigens are not specific to this capsular group and could protect against other meningococcal capsular groups that share the same antigens as those in the vaccine. Infants and toddlers immunized with MenB vaccine are expected to develop bactericidal antibodies against ST11 MenW. Data on age distribution suggest that ≈70% of MenW cases in infants could be prevented by MenB vaccination at 2 and 4 months of age. Beginning in mid-2016, the MenB vaccine booster for children 1 year of age is also expected to protect toddlers, for whom MenW cases have also rapidly increased (*3*).
